# Supplementary material for: Learning curve and surgical outcome of robotic assisted colorectal surgery with ERAS program
Source: Sci Rep. 2022 Nov 29;12:20566. doi: 10.1038/s41598-022-24665-w (PMC9709162; doi:10.1038/s41598-022-24665-w)
Supplement: Supplementary file 1 — Supplementary Information 1. [file 41598_2022_24665_MOESM1_ESM.zip › LSC Raw data-Ñ[▒K/079/N_ñw╝╨░O▒Kñσ.pdf]

W85 / W85

臺中榮民總醫院

[redacted]

基本資料

- 室別： 開刀房-05室，手術科別： GS+CRS，手術類別： 常規，日期： 2020/05/01
- 進入時間： 0750 ； 麻醉開始時間： 0810 ； Time Out時間： 0940 ； 劃刀時間： 0941 ； 手術結束時間： 2110 送出時間： 2120 ；
- 麻醉方式： GE

  診斷：RS Colon /c Liver mets, S458 xN, S23x5, S7x1. S6x3, deep S6x?

  手術名稱：1.Low anterior resection with colo-anal anastomosis
  2.Laparoscopy
  3.Segemental hepatectomy-three segement
  4.Biliary cholecystectomy
  5.Biliary operative sonogram

  手術局部用藥：nil

  術前預防性抗生素：
  - 抗生素名稱一： Veterin 1GM IVD由麻醉護理師執行
  - 時間： 0935
  - 上次簽章: 邱曉玲

  ，追加預防性抗生素：
  - 抗生素名稱一： Veterin 1GM IVD由麻醉護理師執行
  - 時間： 1335
  - 上次簽章: 劉裴瑀
  - 抗生素名稱二： Veterin 1GM IVD由麻醉護理師執行
  - 時間： 1750
  - 記錄者： 鐘?彧
- 手術醫師： 劉嘯天，賴家鈺，蔡易儒，陳映菁，蔣鋒帆，江世偉，林陳彥，林志安，楊曙榮，林昌霖
- 麻醉醫師： VS 李富榮
-
  - 刷手護士A ： 陳祈安，時間： 0750，接替刷手一： 李濘伭，時間： 1230，接替刷手二： 李濘伭/陳祈安，時間： 1300
  - 流動護士A ： 邱曉玲，時間： 0750，接替流動一： 劉裴瑀，時間： 1200，接替流動二： 虞君璇，時間： 1700
- 更多接替及流動護士： 接替刷手三： 鐘?彧，時間： 1500，接替刷手四： 馬景榆，時間： 1715，接替流動三： 鐘?彧，時間： 1745

皮膚狀況

A-V Shunt： 無

術前： 不完整

- 2.瘀傷

部位(術前)：

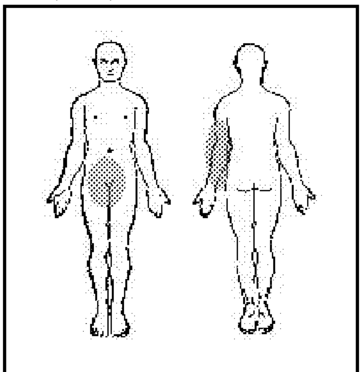

下腹部零星結痂傷口存，左手肘瘀青存

記錄者： 上次簽章: 邱曉玲

術後： 不完整

部位(術後)：

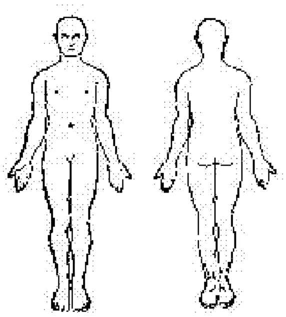

同術前。

記錄者： 鐘?彧

手術準備

手術臥式

-
    - 手術臥式一： 截石臥式
    - 使用附件： 溫毯 38 ℃，頭圈，腳架
    - 皮膚準備： 10%Tr.Betadine，10%Aq Betadine，Hibiscrub
    - 電燒： 使用電燒: 電極片位置： 右大腿，記錄者： 上次簽章: 邱曉玲
-
    - 手術臥式二： 仰臥
    - 使用附件： 溫毯 38 ℃，頭圈，其它： 足踝墊x2.壓克力板
    - 皮膚準備： 10%Tr.Betadine，Hibiscrub
    - 電燒： 使用電燒: 電極片位置： 左大腿，記錄者： 鐘?彧

止血帶T： 無

植入物

- 植入物名稱/號碼/數量/部位： Ethicon Endo GIA 45x4.8mmx3.CDH 29mmx1 /S-colon

, 記錄者： 上次簽章: 邱曉玲

標本送檢

- 細菌培養 無
- 細胞學 無
- 冰凍切片 無
- 病理標本
  組織名稱一：S colon
  ,
  組織名稱二：distal cut end
  ,
  組織名稱三：IMA root
  , 更多組織名稱
    - 組織名稱四：LL1
    - 組織名稱五：LL2
    - 組織名稱六：LL3
    - 組織名稱七：LL4
    - 組織名稱八：LL5
    - 組織名稱九：LL6
    - 組織名稱十：S6-1

  , 更多組織名稱
    - 組織名稱十一：S6-2
    - 組織名稱十二：S6-3
    - 組織名稱十三：S6-4
    - 組織名稱十四：S6-5
    - 組織名稱十五：S4
    - 組織名稱十六：S7

- ○ 組織名稱十七： S4,5,8+GB
- 截肢 無

標本交付

-
    - ○ 標本： 3 袋
    - ○ 標本： 0 盒
    - ○ 標本： 0 瓶
    - ○ 細胞學： 0 管
    - ○ Culture Tube： 0 管
    - ○ 被交付醫師： CR 林昌霖
    - ○
        - ■ 記錄者： 鐘?彧
        - ■ 時間： 2100
-
    - ○ 標本： 14 袋
    - ○ 標本： 0 盒
    - ○ 標本： 0 瓶
    - ○ 細胞學： 0 管
    - ○ Culture Tube： 0 管
    - ○ 被交付醫師： CR 林昌霖
    - ○
        - ■ 記錄者： 鐘?彧
        - ■ 時間： 2100

傷口術後敷料

傷口敷料： 傷口敷料： 無

- 請於圖上標示部位：

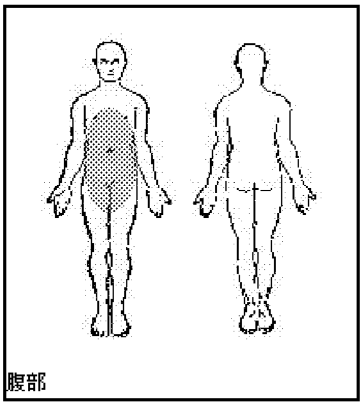

- 傷口敷料： 紗布

導管

導管	天數	導管資訊	插入日期	插入時間	更換日期	拔除日期	拔除時間	拔除原因	預計拔除/更換	預計拔除/更換日期
Sump NG Tube(1)	1	號碼:16 Fr.; 深度:60公分; 部位:胃;	2020/05/01	0840						
Double Lumen CVC (1)	1	號碼:8 Fr.; 深度:外露5cm; 部位:jugular (左); 象限:LU;	2020/05/01	0925						
Endotracheal Tube(1)	1	號碼:6.5 mm.; 深度:21cm; 部位:氣管;	2020/05/01	0815						
Hemo Vac(1)	1	號碼:19 Fr.; 部位:骨盆腔; 註記:左腹;	2020/05/01	1210						
Foley(1)	1	號碼:16 Fr.; 部位:膀胱;	2020/05/01	0835						
Hemo Vac(1)	1	號碼:15 Fr.; 部位:肝下; 註記:右腹內側;	2020/05/01	2005						
Hemo Vac(1)	1	號碼:15 Fr.; 部位:肝上; 註記:右腹外側;	2020/05/01	2010						

Epidural PCA(1)	1	部位:硬脊膜外;	2020/05/01	0820						
A-line(1)	1	號碼:20 G; 部位:左手;	2020/05/01	0830						
Hemo Vac(1)	1	號碼:15 Fr.; 部位:肝下; 註記:左腹;	2020/05/01	2000						

用物計數

- 手術中出血量： 690 c.c.
- 手術中尿量： 1200 c.c.
-
  - 其它： 無

用物計數：

- 部位一： Rectum，1.器械： 正確，2.縫針： 正確，3.刀片： 正確，4.紗布： 正確，5.腹部墊： 未使用，6.紗球： 未使用，7.PEANUTS： 未使用，8.紗條： 未使用，9.絲帶： 未使用，10.針頭： 未使用，11.Vessel loop： 未使用，12.Boots： 未使用，13.Jelco： 未使用，14.棉條： 未使用，
  - 第一次：
    - 刷手護士 ： 陳祈安
    - 流動護士： 上次簽章: 邱曉玲
    - 時間： 1150

  ,
  - 第二次：
    - 刷手護士 ： 陳祈安
    - 流動護士： 上次簽章: 劉裴瑀
    - 時間： 1210

  ,
  - 第三次：
    - 刷手護士 ： 陳祈安
    - 流動護士： 上次簽章: 劉裴瑀
    - 時間： 1220
- 部位二： Liver，
  - 第一次：
    - 刷手護士 ： 馬景榆
    - 流動護士： 鐘?彧
    - 時間： 2020

  ,
  - 第二次：
    - 刷手護士 ： 馬景榆
    - 流動護士： 鐘?彧
    - 時間： 2040

  ,
  - 第三次：
    - 刷手護士 ： 馬景榆
    - 流動護士： 鐘?彧
    - 時間： 2100
- 部位三： NIL，
  - 第一次：
    - 流動護士：

  ,
  - 第二次：
    - 流動護士：

  ,
  - 第三次：
    - 流動護士：

備註

備註： 切肝開始:14點16分
肝下時間:19點25分
肝重:410克
肝缺血:2時44分11秒，共夾17次

- 記錄者： 鐘?彧
- 時間： 2120

填寫護理人員 鐘?彧

正式摘要

W85 / W85

doctorRank:B
